# Population Pharmacokinetics and Model-Based Dosing Optimization of Teicoplanin in Pediatric Patients

**DOI:** 10.3389/fphar.2020.594562

**Published:** 2020-12-08

**Authors:** Tao Zhang, Dan Sun, Zuocheng Shu, Ziyun Duan, Yang Liu, Qian Du, Ying Zhang, Yuzhu Dong, Taotao Wang, Sasa Hu, Hua Cheng, Yalin Dong

**Affiliations:** ^1^ Department of Pharmacy, The First Affiliated Hospital of Xi’an Jiaotong University, Xi’an, China; ^2^ Department of Pharmacy, The Affiliated Children Hospital of Xi’an Jiaotong University, Xi’an, China

**Keywords:** teicoplanin, pediatrics, population pharmacokinetics, dosing optimization, Monte Carlo simulation

## Abstract

**Objectives:** The pharmacokinetics (PK) of teicoplanin differs in children compared with adults. Our aim was to determine the PK of teicoplanin in an Asian pediatric population and to optimize dosage regimens.

**Methods:** This was a retrospective PK study and all the data were collected from hospitalized children. We developed a population PK model using sparse data, and Monte Carlo simulation was used to assess the ability of standard teicoplanin regimen and other different dosage regimens. The optimal dosing regimens were defined as achieving the target trough concentration (*C*_min_) of 10 mg/L and pharmacokinetic/pharmacodynamic (PK/PD, [AUC_24_/MIC]) of 125 for moderate infection. For severe infection, the optimal dosing regimens were defined as achieving the target 15 mg/L and AUC_24_/MIC of 345.

**Results:** 159 children were included and 1.5 samples/children on average were provided. Estimated clearance of teicoplanin was 0.694 L/h (0.784/L/h/70 kg) and volume of distribution was 1.39 L. Teicoplanin standard loading dose was adequate for moderate infection, while 13 mg/kg was needed for severer infection. With standard maintenance doses, both patients with moderate and severe infection failed to achieve the target *C*_min_. 12 and 16 mg/kg/day were required to achieve a *C*_min_ ≥ 10 and 15 mg/L, respectively. However, standard maintenance dose was adequate to achieve AUC_24_/MIC ≥ 125 for moderate infection, and 12 mg/kg/day was needed to achieve AUC_24_/MIC ≥ 345 for severe infection. Lower weight and serum creatinine were associated with higher dose.

**Conclusion:** Optimal doses based on the target *C*_min_ were higher than that based on the PK/PD target. To achieve the *C*_min_ and PK/PD targets simultaneously, a standard loading dose was adequate for moderate infection based on simulation, while dosing higher than standard doses were required in other situation. Further clinical studies with rich sampling from children is required to confirm our findings.

## Introduction

Teicoplanin is a glycopeptide antibiotic with activity against methicillin-resistant *Staphylococcus aureus* (MRSA) (Traina and Bonati, 1984). The marketed drug is hydrophilic predominantly bound to albumin in plasma (>90%) (Lukas et al., 2004) and has a longer elimination half-life than vancomycin (Kasai et al., 2018). Teicoplanin trough concentration (*C*_min_) is closely associated with clinical efficacy. For the moderate (such as respiratory tract infections, urinary tract infections and skin and soft-tissue infections) and severe infection (such as sepsis, infective endocarditis, bone and joint infections), *C*_min_ of at least 10 and 15 mg/L are recommended, respectively (British Medical Association, 2015-2016). However, the standard dosage regimens appear to be inconsistent with the emerging scientific evidence. In previous clinical studies, the proportion of children failing to achieve the target *C*_min_ were 48–89% (Sanchez et al., 1999; Strenger et al., 2013; Zhao et al., 2015). The mean *C*_min_ of teicoplanin were 4.8/5.7/5.9 mg/L at 24/72/168 h, respectively, after the first dose (Sanchez et al., 1999). Even though higher doses were prescribed, 14.1% still had *C*_min_ <10 mg/L (Strenger et al., 2013), and the overall mean *C*_min_ was 9.0 mg/L (Lukas et al., 2004). Yet, the optimal dose of teicoplanin remains to be determined.

Antibiotic dosing determined by pharmacokinetics/pharmacodynamics (PK/PD) data also has been recommended (Kalil et al., 2016). The index that best correlates with teicoplanin antibacterial activity is the ratio of 24-h area under the concentration-time curve to the minimum inhibitory concentration (AUC_24_/MIC) (Ramos‐Martin et al., 2017a). AUC_24_/MIC goals of ≥ 125 and 345 could predict successful outcomes for moderate and severe infection, respectively (Kuti et al., 2008; Ahn et al., 2011). To date, no data has provided a comprehensive understanding the ability of standard dosage regimens of teicoplanin to achieve the suggested PK/PD targets in children.

Previous studies investigated the impact of covariates on pharmacokinetics of teicoplanin in children. A trend of clearance decreasing with increasing age has been observed (Reed et al., 1997). It is considered to be at high risk of PK variability because less fat, higher volume of water and immature renal function in neonate and infant (<1 year) (Friis-Hansen, 1971), especially in the presence of various pathophysiological conditions such as sepsis, fluid overload, effusions, hypoalbuminaemia, and altered renal function, making drug dosing requirements can be difficult to predict. Moreover, it has been demonstrated that nearly 60% of children in pediatric intensive care unit (PICU) exhibit augmented renal clearance (ARC), resulting in low drug exposure due to enhanced excretion (Van Der Heggen et al., 2019). Little is known about the PK of teicoplanin in children (eight studies in total), which greatly hinder the dosing optimization of teicoplanin in children, and only one of them involves Asian children (Supplementary Table S1) (Terragna et al., 1988; Reed et al., 1997; Aarons et al., 1998; Sanchez et al., 1999; Lukas et al., 2004; Ramos-Martin et al., 2014; Zhao et al., 2015; Gao et al., 2020). The objectives of this analysis were to: 1) determine the PK of teicoplanin in Asian children by using a population approach; 2) evaluate the standard dosage regimens of teicoplanin; and 3) establish a simulation-based dosage regimens in this vulnerable population.

## Methods

### Study Design and Patient Population

This was a retrospective PK study performed in two hospitals in China according to the principles of the current Declaration of Helsinki and Good Clinical Practice (Hospital 1: the First Affiliated Hospital of Xi’an Jiaotong University; Hospital 2: the Affiliated Children Hospital of Xi’an Jiaotong University). The protocol was approved by the institutional review board of each study site (No.XJTU1AF2017LSK-28). All patients aged 1 month to 18 years old receiving teicoplanin (Targocid, Sanofi-Aventis) for proven or suspected MRSA infection were selected for the study over 33-month period (March 2017 and November 2019). Children were excluded if a complete teicoplanin dosing history or precise sampling time was not available. The demographic variables with potential impact on the PK of teicoplanin and details of teicoplanin administration (dose and infusion start and stop times) were extracted from medical records retrospectively by a trained research assistant. If serum creatinine (SCr) readings were unavailable around the teicoplanin dosing (±48 h), the closest available SCr reading would be imputed. Creatinine clearance (CLcr) was estimated by Cockcroft formula: CLcr = (140 – age (years)) ×weight (WT, kg) × 0.85 (if female)/0.818 × SCr (μmol/L), instead of Schwartz formula due to the lack of height data in most children (Cockcroft and Gault, 1976).

### Teicoplanin Dosing, Blood Sampling, and Measurement

Teicoplanin was administered at three loading doses of 10 mg/kg every 12 h, followed by 6–10 mg/kg/day. Types of blood samples included therapeutic drug monitoring (TDM) sample, and opportunistic sample. TDM was typically performed within 30 min preceding a dose at steady state. Samples were centrifuged for 10 min. Serum was separated and stored at −80°C until analysis. The laboratory staff were allowed to identify the opportunistic samples with the timings of blood taking documented and store them at −80°C after routine testing and pretreatment. Teicoplanin concentrations were determined with a validated high performance liquid chromatography method. The calibration curve ranged from 2.5 to 100 mg/L, and lower limit of detection (LLOQ) of this assay was 2.5 mg/L. Intra- and inter-day precision values were 3.5 and 6.2%, respectively (Wang et al., 2015). For the samples below the LLOQ, concentration values were recorded as LLOQ of 2.5 mg/L.

### Population Pharmacokinetic Analysis

Population pharmacokinetic (PPK) analysis was performed using NONMEM (version 7.2). A one-compartment PK model with first-order elimination (ADVAN1 TRANS2) was implemented. The concentration-time data for teicoplanin were modeled by first-order conditional estimation with interaction (FOCE-I). We evaluated inter-individual variability using an exponential error model. Residual variability was selected from additive, proportional, exponential, and combined additive and proportional error models according to acceptable standard errors, physiological plausibility of population clearance (CL) and distribution volume (V_d_) estimates, improvement of the objective function value (OFV) and good visual representation of standard diagnostic plots. Demographic characteristics (age, gender, WT), renal functions (blood urea nitrogen, SCr, CLcr), biochemical data (total protein, albumin), status of disease (sepsis, endocarditis), and nephrotoxic medications received during teicoplanin therapy were investigated as potential variables on PK parameters. CLcr was calculated by the Cockcroft formula (Cockcroft and Gault, 1976). A covariate model was developed using a standard stepwise forward-addition backward deletion procedure to ascertain the statistical significance of each covariate. The effects of continuous covariates were modeled using linear, power and exponential models. For categorical covariates, the effect on PK parameter was described by an exponential model. During forward selection, a covariate would be retained if a decrease in objective function value (OFV) was > 3.84 [*p* < 0.05, χ^2^ distribution, degree of freedom (df) = 1] after addition to the basic model, and then all the covariates selected were added simultaneously into a full model. A more stringent criterion was used for the backward elimination step, where a covariate was independently removed from the full model if the increase in OFV was < 10.83 (*p* < 0.001, χ^2^ distribution, df = 1). If the 95% confidence interval of the covariate coefficient included zero, the particular form was rejected.

### Model Evaluation

Evaluation of the model was first based on goodness-of-fit plots. To evaluate the accuracy and stability of the final model, a bootstrap, normalized prediction distribution errors and visual predictive checking (VPC) were performed (PsN). Additionally, the predictive performance of the final model was externally evaluated in a separate patient cohort by calculating the prediction error (PE) and absolute prediction error (APE). The separate patient cohort and patients used for model development come from the same two hospitals. The model with PE value within ± 15% and ± 20% for concentration ≥ 10 and < 10 mg/L, respectively, were considered acceptable. PE and APE are calculated by the following equations (Menichetti et al., 1994; Svetitsky et al., 2009).

$$\text{PE}=\frac{\text{Model}\;\text{predicted}\;\text{concentration}-\text{Observed}\;\text{concentration}}{\text{Observed}\;\text{concentration}}\times 100\%$$

$$\text{APE}=\frac{\left|\text{Model}\;\text{predicted}\;\text{concentration}-\text{Observed}\;\text{concentration}\right|}{\text{Observed}\;\text{concentration}}\times 100\%$$

### Simulation of Dosage Regimens

Monte Carlo simulations were performed to generate 5,000 virtual children. The PK parameters obtained from final model of each patient were used to predict the concentration-time profiles for different teicoplanin weight-based loading and maintenance dosage regimens. Three loading doses were simulated and *C*_min_ were predicted by the day 3 of therapy. *C*_min_ at steady state was predicted for maintenance dosing (by the day 5). A dosage regimen was defined as optimal if mean *C*_min_ reaches 10 and 15 mg/L for moderate and severe infection, respectively. The proportion of patients with potentially toxic concentration (>60 mg/L) were also calculated (Ramos‐Martin et al., 2017b).

Based on the discrete MIC distributions for the MRSA released by the European Committee on Antimicrobial Susceptibility Testing (0.032–16 mg/L, https://mic.eucast.org/Eucast2/regShow.jsp?Id=20922), the cumulative fraction of response (CFR) was also calculated as the weighted average of the probability of target attainment across the MIC strata to define the optimal dosage regimens able to attain the AUC_24_/MIC target of 125 and 345. AUC_24_ was calculated in this study by the formula: AUC_24_ = Daily Dose/CL, which refers to the AUC at steady state. A CFR value of ≥ 90% was considered to be the minimum for achieving optimal empirical therapy (Masterton et al., 2005).

## Results

### Patient Population

**FIGURE 1:**
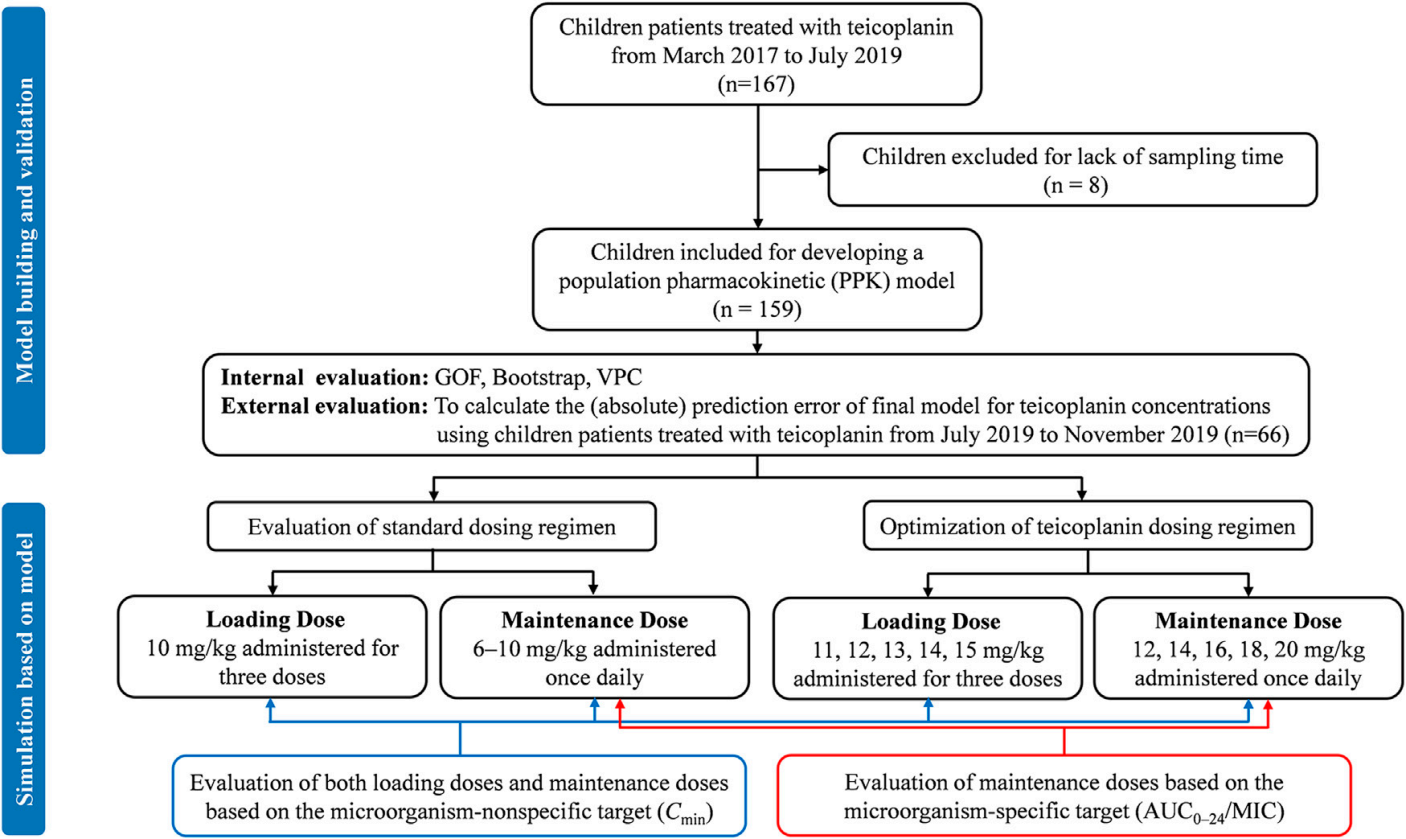
Study flow chart and simulation workflow. GOF, goodness-of-fit; VPC, visual predictive check.

An overview of the entire study flow chart is shown in Figure 1. After excluding eight patients due to lack of sampling time, 159 children with 236 drug concentrations were included for model development eventually. The demographics and clinical characteristics are summarized in Table 1; Supplementary Table S2. Out of the 236 teicoplanin concentrations, 212 (89.8%) were drawn for TDM. Six plasma concentrations fell below the LLOD. 12 (5.1%) had imputed SCr readings. Nine and four children from Hospital 1 were included in model-building and evaluation, respectively. Nine children developed nephrotoxicity during hospitalization and all of them occurred this after the last sample was collected.

**TABLE 1:** Demographic and clinical information for all patients included in model building and evaluation analysis.

Patient characteristic	Values	
	Model-building data (*n* = 159)	Model evaluation data (*n* = 66)
Samplings	236	89
Male/female patients (n, %)	87 (54.7)/72 (45.3)	38 (57.6)/28 (42.4)
Age (yr)	4.1 ± 3.4 (3.7, 0.2–14.0)	4.6 ± 3.8 (3.8, 0.2–13.7)
Patients aged (n, %)	—	—
<2	51 (32.1)	20 (30.3)
2–10	98 (61.6)	37 (56.1)
≥10	10 (6.3)	9 (13.6)
Weight (kg)	16.7 ± 10.1 (14.8, 2.9–69.0)	17.9 ± 12.1 (16.0, 3.0–67.0)
Serum creatinine concentration (μmol/L)	29.1 ± 17.3 (26.0, 10.0–139.0)	25.5 ± 20.1 (22.0, 11.0–176.0)
Creatinine clearance (ml/min)^a^	87.8 ± 47.2 (89.6, 11.0–295.5)	98.0 ± 34.3 (94.9, 11.9–190.4)
Antibiotic indication (n, %)	—	—
Sepsis	39 (24.5)	18 (27.3)
Respiratory tract infection	155 (97.5)	45 (68.2)
Bacteremia	20 (12.6)	10 (15.2)
Bone and joint infection	11 (6.9)	20 (30.3)
Comorbidities (n, %)	—	—
Congenital heart disease	24 (15.1)	6 (9.1)
Myocardial injury	22 (13.8)	1 (1.5)
Malignant hematological disease	91 (57.2)	36 (54.5)
Ventilation (n, %)	48 (30.2)	19 (28.8)
Intensive care unit admissions (n, %)	40 (25.2)	19 (28.8)
Co-medicated with other anti-bacterial drugs (n, %)^b^	—	—
Ceftriaxone	68 (42.8)	12 (18.2)
Meropenem	54 (34.0)	16 (18.2)
Imipenem-cilastatin	72 (45.3)	20 (30.3)
Cefoperazone-sulbactam	31 (19.5)	10 (15.2)
Co-medicated with loop diuretic (n, %)	68 (42.8)	16 (24.2)
Pathogens (n, %)	—	—
*Staphylococcus aureus*	2 (1.3)	3 (1.9)
methicillin-Resistant *Staphylococcus aureus*	6 (3.8)	2 (1.3)
*Staphylococcus epidermidis*	6 (3.8)	1 (0.6)
*E. faecalis*	4 (2.5)	0
*E*. *faecium*	7 (4.4)	0
Teicoplanin loading dose (mg/kg)^c^	9.8 ± 1.4 (10.0, 5.2–16.0)	9.8 ± 1.5 (10.0, 3.0–14.3)
Teicoplanin daily maintenance dose (mg/kg)	9.5 ± 1.2 (10.0, 5.2–12.9)	9.6 ± 1.9 (10.0, 3.7–12.3)
Teicoplanin concentration (mg/L)	8.6 ± 12.1 (10.3, 2.5–82.3)	9.6 ± 5.6 (8.6, 2.5–29.5)

Data are expressed as n (%) or mean ± standard deviation unless specified otherwise.

a Creatinine clearance was calculated by the Cockcroft formula.

b The number of patients co-medicated with at least one other anti-bacterial drug were summarized.

c Administered for three doses at the start of teicoplanin therapy.

### Population Pharmacokinetic Analysis and Model Evaluation

A one-compartment PPK model with an exponential error model for inter-individual variability and additive error model for residual variability resulted in the lowest in OFV for the base model. In the final PK model (OFV = 971.014), WT and SCr were identified as significant covariates for CL, while the OFV of a reduced model without this WT or SCr increased to 1067.599 and 971.000, respectively. WT was also a significant covariate for V_d_, while the OFV of a reduced model without WT on V_d_ increased to 987.532. Supplementary Table S3 summarizes details of the model development process and the population values for CL and V_d_ are derived as follows:

$$CL\;\left(L/h\right)=0.0694\times \left(1+\theta_{1}\times \frac{WT}{16.71}\right)\times \theta_{2}^{\left(SCr/29.075\right)}\times e^{\eta 1}$$

$$V_{d}\left(L\right)=1.39\times \theta_{3}^{\left(WT/16.71\right)}\times e^{\eta 2}$$

The coefficient of variation decreased from 123.3% to 65.9% for CL and from 128.1% to 61.0% for V_d_ after adding the covariates, indicating that the final model accounts for 46.6% and 52.4% of the variability of CL and V_d_ in the data, respectively. The shrinkage were 26.9% and 19.8% for CL and V_d_, respectively, and 24.4% for residual error.

**FIGURE 2:**
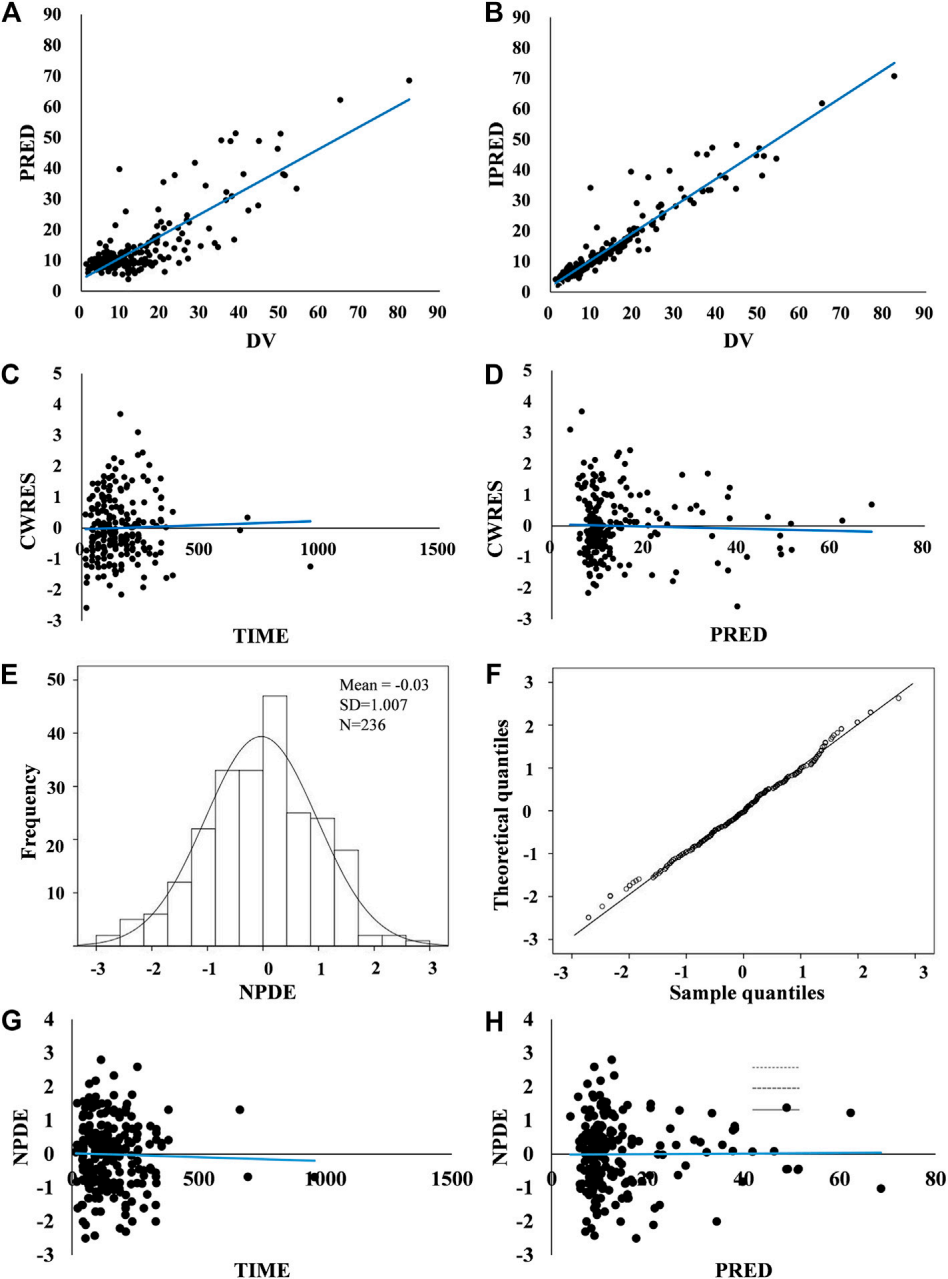
Model evaluation. **(A,B)** Routine diagnostic goodness-of-fit plots: population predicted (PRED) vs. observed concentrations (DV) and individual predicted (IPRED) vs. observed concentrations (DV). **(C,D)** Conditional weighted residuals (CWRES) vs. time and conditional weighted residuals (CWRES) vs. population predicted concentrations (PRED). A solid blue line indicates a trend line. Standard goodness-of-fit of the model showed no obvious systematic bias. There were no trends in conditional weighted residuals distributions. **(E–H)** Normalized prediction distribution errors (NPDE): Q-Q plot of the distribution of the NPDE vs. the theoretical N–(0, 1) distribution and a histogram of the distribution of the NPDE, with the density of the standard Gaussian distribution overlaid. NPDE distribution with the mean of 0.03 met well the theoretical N – (0, 1) distribution, and no trend in the scatterplots was observed, indicating that the fit of the model to the data was acceptable.

**FIGURE 3:**
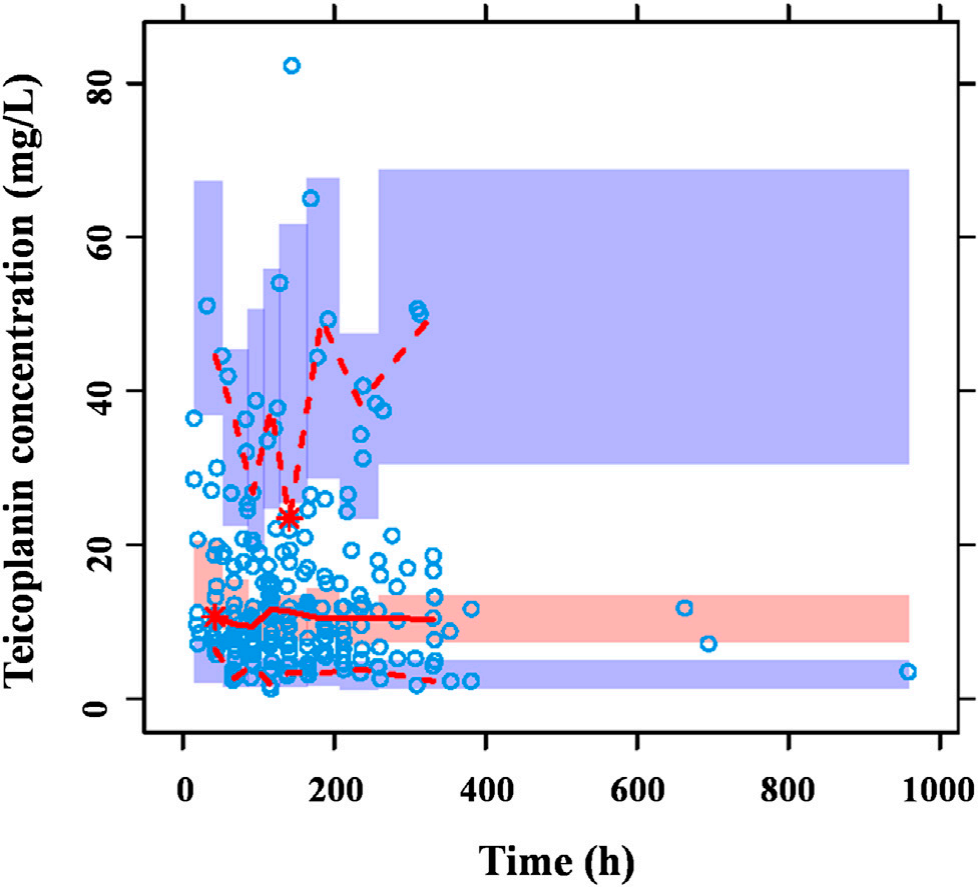
Prediction-corrected VPC generated from a Monte Carlo simulation (*n* = 1,000) for patients used in model development. The blue circles represent the prediction-corrected observed concentrations. The red solid line represents the median prediction-corrected observed concentrations and pink field represents simulation-based 95% confidence intervals for the median. The observed 5% and 95% percentiles are presented with red dashed lines and the 95% intervals for the model-predicted percentiles are shown as corresponding purple fields. VPC demonstrated that 90.7% observations fell within the 90% prediction interval of simulated concentrations out of 1,000 simulated data sets, indicating that the model-based simulated quantities were in good agreement with teicoplanin measured concentration.

Graphical and statistical model evaluation showed well stability and robustness of the final model (Figures 2, 3 and Table 2). The external validation dataset for teicoplanin consisted of 89 concentrations from 66 children with similar demographics to those of the subjects in the PPK analysis (Table 1). The predictive performance was acceptable with a mean PE of −0.24%, and with a mean APE of 10.48%. The percentage of population prediction error within ± 20% for *C*_min_ < 10 mg/L was 94.8% (55/58), and within ±15% for *C*_min_ ≥ 10 mg/L was 89.1% (27/31).

**TABLE 2:** Population pharmacokinetic parameter estimates of final model and bootstrap results from final model.

Parameters	Final model		Estimates based on 1,000 bootstrap replicates^a^	
	Estimate values	Relative standard deviation (%)	Mean	95% confidence interval
CL (L/h)	0.0694	11.3	0.0718	0.0453–0.0983
V_d_ (L)	1.39	11.0	1.77	1.34–2.20
θ_wt_ on CL	2.82	20.6	3.62	1.21–6.03
θ_SCr_ on CL	0.882	5.0	0.794	0.688–0.9
θ_wt_ on V_d_	1.75	6.3	1.76	1.29–2.23
IIV (%)				
CV-CL	65.9	17.6	64.1	57.3–71.9
CV-V_d_	61.0	42.5	69.6	43.8–90.5
Residual variability (%)				
CV-σ	7.0	21.9	8.5	5.1–11.9

Abbreviations: CL, clearance; ^V^_d_, volume of distribution; WT, weight; SCr, serum creatinine; IIV, inter-individual variability; CV, coefficient of variation.

a Bootstrap success rate = 96.5%.

### Simulation of Dosage Regimens

Based on final model, the simulated population was stratified by the various WT and renal function groups to evaluate the effect of these two variates on the optimal dosage regimens. In order to clarify the trend of the effect of SCr on the dosing regimen, the lower limit of SCr range in adult with normal renal function (44 μmol/L) was selected as the typical cut-off value for the simulation due to the lack of standard level of SCr for children.

**FIGURE 4:**
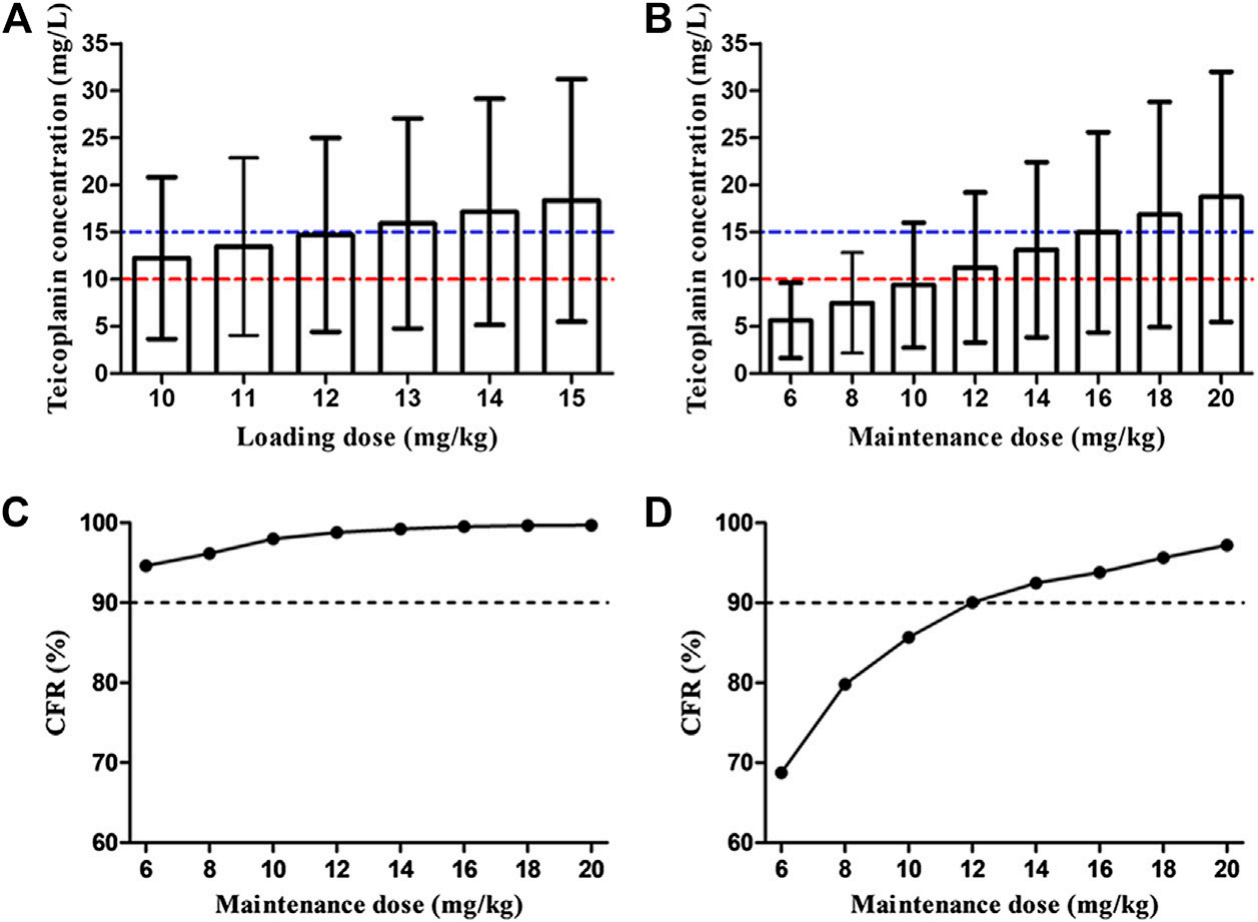
Overall teicoplanin *C*_min_ with different loading doses **(A)** and maintenance doses **(B)**. Each bar represents the mean ± standard deviation. The dashed red line and blue line indicate the targets *C*_min_ of 10 mg/L (moderate infection) and 15 mg/L (severe infection), respectively. Cumulative fraction of response (CFR) of different maintenance doses for AUC_24_/MIC ≥ 125 **(C)** and 345 **(D)**. AUC_24_/MIC ≥ 125 and 345 were defined as the target values for moderate and severe infection, respectively. The MIC range and distribution are based on the EUCAST data published in 2019 (https://mic.eucast.org/Eucast2/regShow.jsp?Id=20922). Loading doses were administered every 12 h for three doses and *C*_min_ was simulated by day 3 (48 h). Maintenance doses were administered once daily and *C*_min_ was simulated by day 5 (96 h).

**FIGURE 5:**
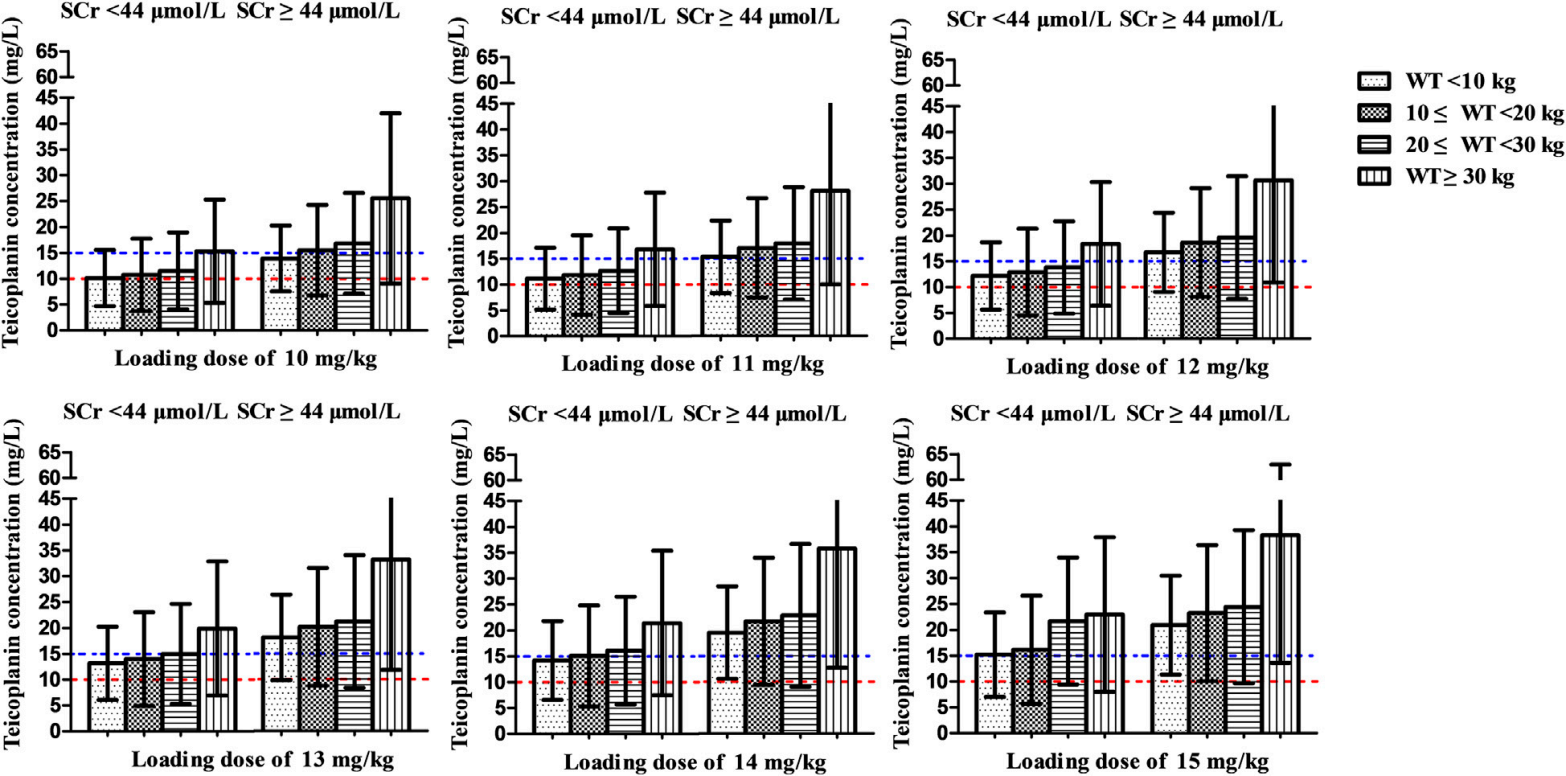
Mean teicoplanin Cmin with different loading doses in subgroups stratified by weight (WT, kg) and serum creatinine (SCr, μmol/L). Each bar represents the mean ± standard deviation. Loading doses were administered every 12 h for three doses and *C*_min_ was simulated by day 3 (48 h). The dashed red line and blue line indicate the target *C*_min_ of 10 mg/L (moderate infection) and 15 mg/L (severe infection), respectively.

Figure 4A shows the mean *C*_min_ achieved with different loading dose regimens. A standard loading dose of 10 mg/kg achieved a mean *C*_min_ of 12.0 mg/L, which is sufficient for moderate infection, while 13 mg/kg (15.6 mg/L) would be effective in achieving mean *C*_min_ of 15 mg/L for severe infection. All the optimal dosage regimens are summarized in Table 3. Higher loading dose correlated with lower WT and SCr according to subgroup analysis (Figure 5).

**TABLE 3:** Optimal dosing regimens achieving target teicoplanin *C*_min_ at 48 h for loading dose regimens and at day 5 for maintenance dose regimens, and AUC_24_/MIC for moderate and severe infection^a^.

Subgroup		Moderate infection			Severe infection		
		*C*_min_ ≥ 10 mg/L		AUC_24_/MIC ≥ 125	*C*_min_ ≥ 15 mg/L		AUC_24_/MIC ≥ 345
WT	SCr	Loading dose	Maintenance dose	Maintenance dose	Loading dose	Maintenance dose	Maintenance dose
<10	<44	10 mg/kg q12h × 3	14 mg/kg q24h	6 mg/kg q24h	15 mg/kg q12h × 3	20 mg/kg q24h	16 mg/kg q24h
	≥44	10 mg/kg q12h × 3	10 mg/kg q24h	6 mg/kg q24h	11 mg/kg q12h × 3	16 mg/kg q24h	14 mg/kg q24h
10 ≤ WT < 20	<44	10 mg/kg q12h × 3	12 mg/kg q24h	6 mg/kg q24h	14 mg/kg q12h × 3	18 mg/kg q24h	12 mg/kg q24h
	≥44	10 mg/kg q12h × 3	10 mg/kg q24h	6 mg/kg q24h	10 mg/kg q12h × 3	14 mg/kg q24h	12 mg/kg q24h
20 ≤ WT < 30	<44	10 mg/kg q12h × 3	12 mg/kg q24h	6 mg/kg q24h	13 mg/kg q12h × 3	18 mg/kg q24h	10 mg/kg q24h
	≥44	10 mg/kg q12h × 3	8 mg/kg q24h	6 mg/kg q24h	10 mg/kg q12h × 3	12 mg/kg q24h	10 mg/kg q24h
WT ≥ 30	<44	10 mg/kg q12h × 3	10 mg/kg q24h	6 mg/kg q24h	10 mg/kg q12h × 3	14 mg/kg q24h	10 mg/kg q24h
	≥44	10 mg/kg q12h × 3	6 mg/kg q24h	6 mg/kg q24h	10 mg/kg q12h × 3	8 mg/kg q24h	10 mg/kg q24h
Overall		10 mg/kg q12h × 3	12 mg/kg q24h	6 mg/kg q24h	13 mg/kg q12h × 3	16 mg/kg q24h	12 mg/kg q24h

Abbreviations: *C*_min,_ trough concentration; WT, weight (kg); SCr, serum creatinine (μmol/L). AUC_24_/MIC, the ratio of the 24-h area under the curve to the minimum inhibitory concentration.

a *C*_min_ ≥10 mg/L and AUC_24_/MIC ≥ 125 were defined as the target values for moderate infection; C_min_ ≥ 15 mg/L and AUC_24_/MIC ≥ 345 were defined as the target values for severe infection.

**FIGURE 6:**
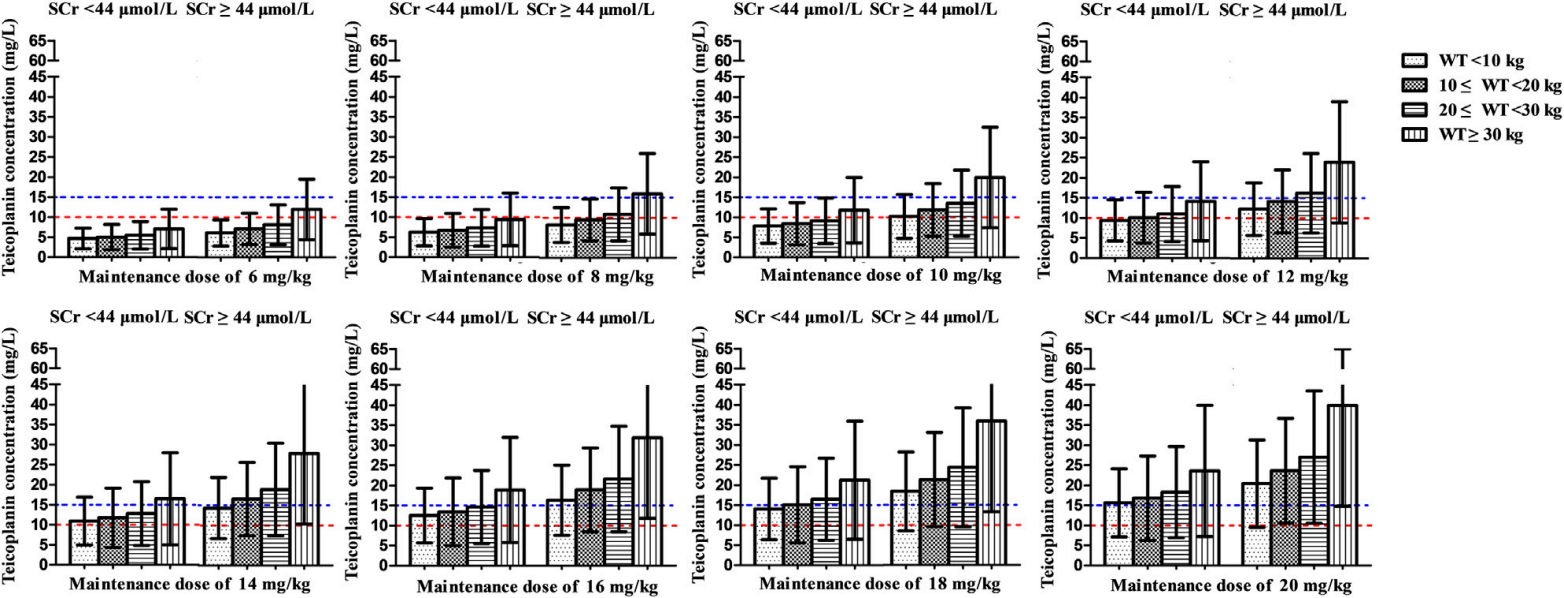
Mean teicoplanin *C*_min_ with different maintenance doses in subgroups stratified by weight (WT, kg) and serum creatinine (SCr, μmol/L). Each bar represents the mean ± standard deviation. Maintenance doses were administered once daily and *C*_min_ was simulated by day 5 (96 h). The dashed red line and blue line indicate the target *C*_min_ of 10 mg/L (moderate infection) and 15 mg/L (severe infection), respectively.

At maintenance doses of 6–10 mg/kg/day proposed by specification, at best, only a mean *C*_min_ of 9.4 mg/L was achieved, which were inadequate both for moderate and severe infection (Figure 4B). 12 and 16 mg/kg/day could achieve mean *C*_min_ of 10 and 15 mg/L, respectively. Higher maintenance doses were required in the patients with lower WT and SCr (Figure 6 and Table 3).

<2% of patients had potentially toxic concentrations (>60 mg/L) across the dosage regimens simulated, indicating that all the dosing strategies involved in our study had acceptable exposures.

**FIGURE 7:**
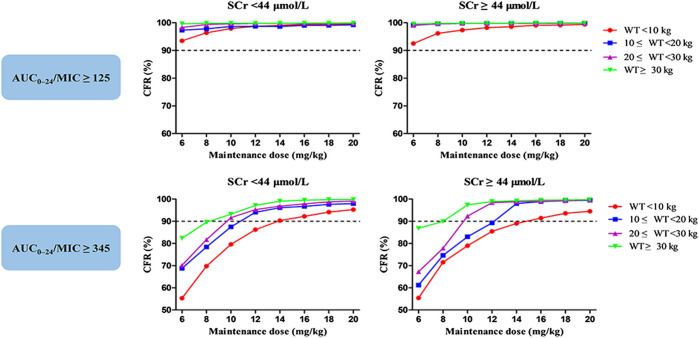
Cumulative fraction of response (CFR) of different maintenance doses for MRSA in subgroups stratified by weight (WT, kg) and serum creatinine (SCr, μmol/L). The pharmacodynamic index was the 24-h area under the plasma concentration–time curve over the minimum inhibitory concentration ratio (AUC_24_/MIC). AUC_24_/MIC ≥ 125 and 345 were defined as the target values for moderate and severe infection, respectively. The MIC range and distribution are based on the EUCAST data published in 2019.

Figures 4C,D display the CFR of different dosage regimens. The standard maintenance doses had overall CFR of 94.6–98.0% for AUC_24_/MIC ≥ 125. However, with an AUC_24_/MIC ≥ 345, only CFR of 68.7–85.7% were obtained. A higher maintenance dose of 12 mg/kg/day achieved a CFR ≥90% for severe infection. In the subgroup analysis, no obvious effect of SCr on the optimal regimens was observed, while maintenance dose presents increase with the decrease of WT in the patients with severe infection (Figure 7).

## Discussion

We developed a PPK model of teicoplanin in Asian children. A highlight in this study is that dosing regimens in children were first optimized using two methods, providing two sets of optimal dosing regimens. On the one hand, the advantage of such way was to compare the results directly from two kind of targets widely adopted in dosing optimization, and understand the differences between them. We deed found that optimal doses based on the target *C*_min_ were higher than that based on the PK/PD target. On the other hand, it is helpful for clinicians and pharmacists to determine the optimal dosing regimens, avoiding the doubts about which optimal dosing regimens are reliable. According our simulation, doses higher than currently recommended in children should be used to achieve both targets of *C*_min_ and PK/PD.

This is the largest PK study of teicoplanin in children (Supplementary Table S1). The covariate analysis revealed that WT and SCr were the significant covariates influencing teicoplanin PK, accounting for around 50% of the observed PK variability, which is higher than other PPK studies in children and adults (Byrne et al., 2015; Ramos-Martin et al., 2017; Zhao et al., 2015). CLcr of children is likely to be overestimated due to young age and small body weight when estimated by Cockcroft formula, and this might be the main reason why the CLcr showed no significant influence on PK parameters of teicoplanin in our study (Cockcroft and Gault, 1976).

Great variation for PK parameters of teicoplanin was presented in children. The typical population values of CL in our study (0.014 L/h/kg) was similar to the range of 0.015–0.024 L/h/kg reported in non-PICU Caucasians previously, but lower than that in PICU Caucasians (0.03–0.074 L/h/kg) (Aarons et al., 1998; Lukas et al., 2004; Ramos-Martin et al., 2014; Reed et al., 1997; Sanchez et al., 1999; Terragna et al., 1988; Zhao et al., 2015). Due to widespread systemic inflammation, patients may often have an ARC in PICU patients (Van Der Heggen et al., 2019), and increased volume of distribution and drug clearance has been observed for hydrophilic drugs, resulting in sub-therapeutic trough concentrations (Hirai et al., 2016). Consequently, higher doses may be required. Lukas, et al. reported that the typical population values of CL and V_d_ were 0.16 L/h/kg and 2.14 L/h/kg, respectively, which are far higher than results from other studies (Lukas et al., 2004). Consistent with Lukas, two studies were also conducted in patients admitted to the PICU, and reported only 0.045 and 0.03 L/h/kg for CL (Reed et al., 1997; Sanchez et al., 1999). A small sample size in Lukas’s study might be one of the reasons for this difference. CL estimate (0.013 L/h/kg) from a most recent study involved Chinese children is almost equal to ours, while much difference in V_d_ (1.85 L/kg) was showed compared with our and other studies (0.2–1.02 L/kg). The estimate of V_d_ in this study (0.15 L/kg) was closest to that published by Ramos-Martin et al. (0.2 L/kg), which could be explained by the similar patients characteristics between our studies (Ramos-Martin et al., 2014) (Supplementary Table S1). Overall, our study provides an important addition to the PK characteristics of teicoplanin and essential foundation for optimizing teicoplanin dosing regimen in this special population.

Loading dose regimen is necessary to reach the effective drug exposure rapidly (Kollef, 2013). However, the standard loading dose was insufficient for severe infection with a mean *C*_min_ of only 12 mg/L achieved in this study. Sanchez reported that the mean *C*_min_ by 48 h were 4.8 mg/L (Sanchez et al., 1999). With higher loading doses of 10–15 mg/kg, the proportion of children with *C*_min_ of <10 mg/L was 14.4% (Strenger et al., 2013). Higher initial loading dose could provide higher drug exposure at the start of treatment. However, the difference appeared to vanish after 14 days when different loading doses were followed by the same dose administered once daily, illuminating the importance of sufficient maintenance dose (Ahn et al., 2011). Our results showed that the current maintenance doses in children only achieved mean *C*_min_ of 5.6–9.4 mg/L, which are in agreement with the *C*_min_ of 4.8–5.9 mg/L achieved in another study (Sanchez et al., 1999). Although a few studies evaluated teicoplanin standard dosage regimens in children, none of them focused on the probability of target attainment according to PK/PD targets (Reed et al., 1997; Sanchez et al., 1999; Zhao et al., 2015). Interestingly, we found that the current maintenance doses of teicoplanin showed sufficient for moderate infection, but not for severe infection in term of PK/PD targets. In summary, the current dosage regimens are associated with a high risk of underdosing in this particular group of patients, and higher doses are needed to improve the probability to achieve the target of *C*_min_ or PK/PD. Zhao et al. suggested a maintenance dose of 15 mg/kg/day in children (Zhao et al., 2015). Even higher doses of 15–20 mg/kg/day were recommended to assure *C*_min_ above 10 mg/L and all patients attain *C*_min_ > 10 mg/L only when a maintenance dose of 20 mg/kg/day was administrated (Dufort et al., 1996). These findings provide additional support to our results to increase the dose of teicoplanin. Although several other studies did not perform optimization for the teicoplanin dosage regimens, they also proposed that children may require relatively higher doses (Reed et al., 1997; Lukas et al., 2004).

There are large differences in the optimal dosage regimens provided by the two methods (Table 3). Taken together, optimal dosage regimens based on the *C*_min_ targets in our study are recommended, which are three loading doses of 10 mg/kg every 12 h, followed by a maintenance dose of 12 mg/kg/day for *C*_min_ of > 10 mg/L and three loading doses of 13 mg/kg every 12 h, followed by a maintenance dose of 16 mg/kg/day for *C*_min_ of > 15 mg/L. The reasons are as follows: 1) The maintenance dose based on the *C*_min_ targets are higher than that based PK/PD targets. In other words, maintenance dose based on the *C*_min_ targets could achieve both microorganism-nonspecific and microorganism-specific targets simultaneously. It is worthy to be noticed that the two evaluation criteria, mean *C*_min_ of 10 (15) mg/L and AUC_24_/MIC ≥ 125 (345), are not in correspondence. It would be more reasonable to define a dose achieving 90% of patients with a *C*_min_ of 10 (15) mg/L as the optimal dose. However, the proportion of patients achieving the desired exposure is far below 90% both in clinical study (Sanchez et al., 1999; Strenger et al., 2013; Zhao et al., 2015; Sanchez et al., 1999; Strenger et al., 2013; Zhao et al., 2015) and our simulation (Supplementary Figures S1, S2). Increasing the magnitude of doses is always the first step to improve the *C*_min_ target attainment rates in such situation. Gao et al. reported dosing regimens for Chinese pediatrics to achieve the *C*_min_ of > 10 mg/L. Three loading doses of 6–12 mg/kg every 12 h, followed by a maintenance doses of 8–10 mg/kg/day were required, which is similar to three loading doses of 10 mg/kg every 12 h, followed by a maintenance dose of 6–14 mg/kg/day in our study (Gao et al., 2020). 2) Although antibiotic dosing as determined by PK/PD data was suggested, lack of practitioner familiarity, unclear benefit, time allocation and training requirements are the biggest obstacles to make it in clinical practice (Kufel et al., 2019). Considerable extra costs for the levels monitoring using AUC is another dilemma (Meng et al., 2019). Teicoplanin exhibits linear PK (Rowland, 1990) and *C*_min_ correlates with AUC_24_ strongly (Cazaubon et al., 2017; Zhao et al., 2015), which make it possible for *C*_min_ as a surrogate of AUC_24_. In the present study, the mean *C*_min_ increased 1.2 and 0.9 mg/L with each 1 mg/kg increase in loading and maintenance dose, respectively. However, the necessity of TDM for teicoplanin is still controversial. TDM for teicoplanin is not performed routinely in clinical practice (Darley and MacGowan, 2004). Even so, exposure control to maximize efficacy should not be neglected and the relatively higher pediatric PK variability supports the use of routine TDM to reduce the risk of clinical failure and the development of drug resistance due to suboptimal drug exposure. Therefore, the situation of low teicoplanin concentration in children is the predominant argument for the routine monitoring of teicoplanin concentrations. A retrospective analysis over a 13 year period indicated that the TDM of teicoplanin has been paid more attention and played an important role in improving the *C*_min_ target attainment rate (Tobin et al., 2010). 3) Children have demonstrated a higher CL of teicoplanin than adults (Rowland, 1990; Tarral et al., 1988). In the adults study published previously, seven out of ten of the teicoplanin CL reported were lower than 0.01 L/h/kg (Byrne et al., 2018; Cazaubon et al., 2017; Kasai et al., 2018; Lamont et al., 2005; Soy et al., 2006; Yamada et al., 2012; Yu et al., 1995), which is similar with that in the normal healthy male volunteers (Thompson et al., 1992) and lower than that in children (0.015–0.074 L/h/kg) (Aarons et al., 1998; Lukas et al., 2004; Ramos-Martin et al., 2014; Reed et al., 1997; Sanchez et al., 1999; Terragna et al., 1988; Zhao et al., 2015). The standard doses for adult were lower compared to that for children before the update of teicoplanin information form (3–6 mg/kg vs. 6–10 mg/kg). However, the standard doses for adult has been increased to 2-fold, but no modification was made for pediatrics (Supplementary Table S4). In fact, the standard doses are not only insufficient for adults (Brink et al., 2008; Matsumoto et al., 2010; Kato et al., 2016), but also for children (Sanchez et al., 1999; Lukas et al., 2004; Strenger et al., 2013; Zhao et al., 2015). 4) Teicoplanin is associated with a lower adverse event compared with vancomycin (Svetitsky et al., 2009) and the proportion of patients achieving *C*_min_ ≥ 60 mg/L is < 2%, showing well safety of all doses simulated. Although nephrotoxicity, hepatotoxicity and drug fever have been reported previously in adults (Greenberg, 1990; Kato et al., 2016), whether higher doses for children would lead to safety concern is still not determined, which remind us to closely monitor the adverse reaction induced by teicoplanin when higher doses are administered.

There are some limitations of this study. First, sparse sampling is not an optimal but very useful method to determine the PK characteristic of drugs in pediatric populations. Although the current final PPK model was developed based on the biggest sample size so far, only 1.5 samples/children on average was provided due to practical reasons. Caution needs to be exercised when interpreting our results in this very variable population. Second, the evaluation and optimization of loading doses were conducted only based on the *C*_min_ targets. The formula used for calculating AUC_24_ is unable to calculate it in a specific period, not like the integral method used by other researchers (Byrne et al., 2017; Cazaubon et al., 2017). However, it could be speculated that the loading doses based on the *C*_min_ targets might obtain sufficient for achievement of the PK/PD target due to lower maintenance doses based on the PK/PD targets. Third, AUC_24_/MIC goals of ≥ 125 and 345, two PK/PD indexes of teicoplanin for efficacy, were used in this study. Additional PK/PD indexes also have been reported, such as 750, 900, and 1800 (Rose et al., 2008; Kanazawa et al., 2011; Matsumoto et al., 2016). Considering that there is not enough evidence to support the correlation of efficacy with 750, 900, and 1800 is suggested to prevent the teicoplanin-resistant *S. aureus*, these target PK/PD ratio were not adopted. We did not evaluate the correlation of AUC_24_/MIC or *C*_min_ with efficiency, because 78% of children had microbial culture results but no specific MIC values and this study was not designed to relate efficacy indicators to clinical outcomes. However, the teicoplanin *C*_min_ and PK/PD targets of children are referred to that for adults, which are largely based on retrospectively studies (Kuti et al., 2008; Ramos-Martin et al., 2017). Other research efforts should evaluate whether these targets could be extrapolated to pediatric patients and compare the AUC_24_/MIC methodology with trough measurement in children.

## Conclusion

In conclusion, we successfully developed and externally validated a PPK model for teicoplanin based on a large cohort of Asian pediatric patients. Under standard protocol, the expected *C*_min_ for children might be undertherapeutic, especially for the children with lower WT and SCr. Dosage regimens of three loading doses of 10/13 mg/kg every 12 h, followed by 12/16 mg/kg/day for moderate/severe infection, respectively, might be required in this particular patient population. Additional well-designed prospective studies with intensive sampling strategy are warranted to evaluate the potential clinical outcome and safety of these optimized dosage regimens.

## Data Availability Statement

The raw data supporting the conclusions of this article will be made available by the authors, without undue reservation.

## Ethics Statement

The studies involving human participants were reviewed and approved by Ethics committee of the first affiliated hospital of Xi’an Jiaotong University Ethics committee of the Affiliated Children Hospital of Xi’an Jiaotong University. Written informed consent from the participants’ legal guardian/next of kin was not required to participate in this study in accordance with the national legislation and the institutional requirements.

## Author Contributions

YD and TZ helped design the study. YD, TZ, DS, ZS, and ZD helped conduct the study collected the data. All authors helped analyze and interpret the data, contributed to the preparation of the manuscript, and approved the final manuscript for submission.

## Funding

This work is supported by the Clinical Research Award of the First Affiliated Hospital of Xi’an Jiaotong University (XJTU1AF-CRF-2017-023), key research and development program of Shaanxi province (2019SF-197), key research and development program in Shaanxi province of China (2019ZDLSF01-05), Natural Science Foundation of Shaanxi Province (2019JQ-388), and National Science Fund for Distinguished Young Scholars (71904155).

## Conflict of Interest

The authors declare that the research was conducted in the absence of any commercial or financial relationships that could be construed as a potential conflict of interest.
