# Supplementary material for: Population Pharmacokinetics and Model-Based Dosing Optimization of Teicoplanin in Pediatric Patients
Source: Front Pharmacol. 2020 Dec 8;11:594562. doi: 10.3389/fphar.2020.594562 (PMC7753357; doi:10.3389/fphar.2020.594562)
Supplement: Supplementary file 1 [file table1.docx]

**Supplementary material**

**TABLES**

TABLE S1 Pharmacokinetic Study of teicoplanin in pediatric patients.

| Study (year) | Patients (n) | Samples type (n) | Study type | Location | Administration | Teicoplanin method | Clearance (CL) , L/h | Volume of distribution (V_d_) , L | Citation |
| --- | --- | --- | --- | --- | --- | --- | --- | --- | --- |
| Gao (2020) | Children infected by Gram-positive bacterial; mean age ± SD: 2.19 ± 2.25 year (136) | Steady-state concentrations (96.77%) (155) | Population pharmacokinetics, prospective study | China | Intravenous infusion | HPLC | CL=0.13×(WT/10)^0.74^×(eGFR/118.99)^0.6^  Q=0.23 | V_1_=2.31×(ln WT/2.3) ^0.14^  V_2_=16.19×(WT/10)^0.19^ | ^[^[^1^](#_ENREF_1)^]^ |
| Zhao (2015) | Children with malignant haematological disease; mean age ± SD: 8.4 ± 4.6 year (85) | TDM and random sample (143) | Population pharmacokinetics, prospective study | France | Intravenous infusion for 3-5 min | Quantitative microsphere system technology | CL=0.491×(WT/27.1)^0.75^×(CLcr/179)^0.606^ | V_1_=12.9×(WT/27.1)  V_2_=25.2×(WT/27.1) | ^[^[^2^](#_ENREF_2)^]^ |
| Ramos-Martin (2014) | Children with acute hematogenous osteomyelitis and gram-positive organisms; mean age ± SD: 4.0 ± 4.3 year (39) | Sparse sample：1, 3, 6, 24 h postdose (298) | Population pharmacokinetics, prospective study | England | Intravenous infusion for 5 min | FPIA | CL=0.023×WT | V_d_ =4.138 | ^[^[^3^](#_ENREF_3)^]^ |
| Lukas (2004) | Children admitted to the PICU with gram-positive organisms; mean age ± SD: 3.1 ± 3.0 year (20) | Sparse sample: 1 h before, 1 and 3 h after the 1st, 3rd, and 5th maintenance dose (NA) | Population pharmacokinetics, prospective study | Greece | Intravenous infusion for 1 h | FPIA | Age <12 months:  CL=0.09×(1+WT^0.75^)  Age ≥12 months:  CL=0.29×(1+WT^0.75^) | Age <12 months:  V_d_=1.05×(1+WT^0.75^)  Age ≥12 months:  V_d_ =3.9×(1+WT^0.75^) | ^[^[^4^](#_ENREF_4)^]^ |
| Sanchez (1999) | Children admitted to the PICU with nosocomial infection; age range, 7 days – 12 year (21) | Sparse sample: 0.5, 2, 6, 12, 24, 48, 72, 168 h after first dose (127) | Pharmacokinetics, prospective study | Spain | IV bolus | HPLC | CL=0.045×WT | V_d_ =1.02×WT | ^[^[^5^](#_ENREF_5)^]^ |
| Aarons (1998) | Children at high risk of infections due to Gram-positive bacteria; age range, 3-12 year (37) | Sparse sample: 3, 9, 12, 15, 18, 24, 27, 36, 48 h after first dose (64) | Population pharmacokinetics, prospective study | Italy | Intramuscular injection | Receptor-antibody sandwich assay | CL/F=0.024×WT^a^ | V_d_ /F=0.61×WT^a^ | ^[^[^6^](#_ENREF_6)^]^ |
| Reed (1997) | Children admitted to the PICU; mean age ± SD: 6 ± 3.1 year (12) | Sparse sample (NA) ^b^ | Pharmacokinetics, prospective study | America | Intravenous infusion for 20-30 min | Both microbiology assay and HPLC | Microbiology assay:  CL=0.037×WT  HPLC:  CL=0.03×WT | Microbiology assay:  V_d_ =0.56×WT  HPLC:  V_d_ =0.5×WT | ^[^[^7^](#_ENREF_7)^]^ |
| Terragna (1988) | Children at high risk of infections due to Gram-positive bacteria or for appropriate medical indications; mean age ± SD: 6.8 ± 1.0 year (13) | Sparse sample: 0, 1, 3, 6, 12, 24, 48, 72, 96, 120, 144, 168 and 192 h after first dose (NA) | Pharmacokinetics, prospective study | Italy | Intravenous infusion for 3-5 min | Microbiology assay | Three compartment model:  CL=0.015×WT  Non-compartmental analysis:  CL=0.015×WT | Three compartment model:  V_d_ =0.80×WT  Non-compartmental analysis:  V_d_ =0.83×WT | ^[^[^8^](#_ENREF_7)^]^ |

Abbreviations: NA, not available; SD: standard deviation; TDM: therapeutic drug monitoring; V_1_, central volume of distribution; V_2_, peripheral volume of distribution; FPIA: fluorescence polarization immunoassay; HPLC: high-performance liquid chromatography; WT: body weight, CLcr, creatinine clearance; PICU, pediatric intensive care unit.

^a^ F(bioavailability)~1 as reported by Ripa et al. (1988).

^b^ Samples obtained at time 0, 45 min, 1, 2, 6, 12 and 24 h after the beginning of the first dose infusion; 1, 4 and 8 h after the start of the second dose infusion; 1, 8 and 24 h after the start of the third dose infusion; 1 and 12 h after the start of the fourth dose infusion; and 0, 30 and 45 min and 1, 2, 6, 12 and 24 h after the start of the fifth dose infusion; 36, 60, 84, 108 and 132 h after the start of fifth and last dose infusion.

**References**

[1]. Gao L, Xu H, Ye Q, et al. Population pharmacokinetics and dosage optimization of teicoplanin in children with different renal functions. Frontiers in Pharmacology 2020; 5;11:552-65.

[2]. Zhao W, Zhang D, Storme T, et al. Population pharmacokinetics and dosing optimization of teicoplanin in children with malignant haematological disease. Br J Clin Pharmacol 2015;80:1197-207.

[3]. Ramos-Martin V, Paulus S, Siner S, et al. Population pharmacokinetics of teicoplanin in children. Antimicrob Agents Chemother 2014;58:6920-7.

[4]. Lukas JC, Karikas G, Gazouli M, et al. Pharmacokinetics of teicoplanin in an ICU population of children and infants. Pharm Res 2004;21:2064-71.

[5]. Sanchez A, Lopez-Herce J, Cueto E, et al. Teicoplanin pharmacokinetics in critically ill paediatric patients. J Antimicrob Chemother 1999;44:407-9.

[6]. Aarons L, Rowland M, Khan A, et al. Plasma and tonsillar tissue pharmacokinetics of teicoplanin following intramuscular administration to children. Eur J Pharm Sci 1998;6:265-70.

[7]. Reed MD, Yamashita TS, Myers CM, et al. The pharmacokinetics of teicoplanin in infants and children. J Antimicrob Chemother 1997;39:789-96.

[8]. Terragna A, Ferrea G, Loy A, et al. Pharmacokinetics of teicoplanin in pediatric patients. Antimicrob Agents Chemother 1988;32:1223-6.

TABLE S2 Demographic and clinical information of children from 2 hospitals.

| Patient characteristic | Values | |
| --- | --- | --- |
|  | Hospital 1 (n=13) | Hospital 2 (n=212) |
| Samplings | 21 | 304 |
| Male/female patients (n, %) | 7 (53.8)/6 (46.2) | 118 (55.7)/94 (44.3) |
| Age (yr) | 11.2 ± 1.8 (11.6, 10.0 – 14.0) | 4.0 ± 3.3 (3.8, 0.2 – 13.7) |
| Patients aged (n, %) |  |  |
| <2 | 0 | 71 (33.5) |
| 2–10 | 0 | 135 (63.7) |
| ≥10 | 13 (100) | 6 (2.8) |
| Weight (kg) | 39.8 ± 12.0 (36, 26 – 69.0) | 14.6 ± 7.4 (14.0, 2.9 – 52.0) |
| Serum creatinine concentration (μmol/L) | 42.0 ± 20.1 (36.9, 19.0 – 103.0) | 23.4 ± 16.6 (21.6, 10.0 – 176.0) |
| Creatinine clearance (mL/min) ^a^ | 92.3 ± 27.8 (93.6, 34.9 – 106.4) | 95.1 ± 30.3 (93.4, 11.0 – 295.5) |
| Antibiotic indication (n, %) |  |  |
| Sepsis | 3 (23.1) | 54 (25.5) |
| Respiratory tract infection | 9 (69.2) | 191 (90.1) |
| Bacteremia | 2 (15.4) | 25 (11.8) |
| Bone and joint infection | 3 (23.1) | 28 (13.2) |
| Comorbidities (n, %) |  |  |
| Congenital heart disease | 0 | 30 (14.2) |
| Myocardial injury | 1 (7.7) | 22 (10.4) |
| Malignant haematological disease | 4 (30.8) | 123 (58.0) |
| Ventilation (n, %) | 1 (7.7) | 66 (31.1) |
| Intensive care unit admissions (n, %) | 1 (7.7) | 48 (22.6) |
| Co-medicated with other anti-bacterial drugs (n, %) ^b^ |  |  |
| Ceftriaxone | 4 (30.8) | 76 (35.8) |
| Meropenem | 8 (61.5) | 62 (29.2) |
| Imipenem-cilastatin | 6 (46.2) | 20 (31.1) |
| Cefoperazone-sulbactam | 2 (15.4) | 10 (13.7) |
| Co-medicated with loop diuretic (n, %) | 1 (7.7) | 16 (39.2) |
| Pathogens (n, %) |  |  |
| *Staphylococcus aureus* | 0 | 5 (2.4) |
| methicillin-resistant *Staphylococcus aureus* | 1 (7.7) | 7 (3.3) |
| *Staphylococcus epidermidis* | 0 | 7 (3.3) |
| *E. faecalis* | 0 | 4 (1.9) |
| *E*. *faecium* | 0 | 7 (3.3) |
| Teicoplanin loading dose (mg/kg) ^c^ | 9.8 ± 1.5 (10.0, 6.0 – 12.0) | 9.8 ± 1.5 (10.0, 3.0 – 16.0) |
| Teicoplanin daily maintenance dose (mg/kg) | 9.6 ± 1.1 (10.0, 6.0 – 10.0) | 9.6 ± 2.1 (10.0, 3.7 – 12.9) |
| Teicoplanin Concentration (mg/L) | 9.3 ± 10.1 (9.2, 2.5 – 72.6) | 9.1 ± 8.9 (10.6, 2.5 – 82.3) |

Data are expressed as n (%) or mean ± standard deviation unless specified otherwise.

^a^ Creatinine clearance was calculated by the Cockcroft formula.

^b^ The number of patients co-medicated with at least one other anti-bacterial drug were summarized.

^c^ Administered for three doses at the start of teicoplanin therapy.

TABLE S3 Covariate analysis ^a^.

| Model | Pharmacokinetic model | OFV | Δ(OFV) | *P* value |
| --- | --- | --- | --- | --- |
| 1 | One-compartment model first-order elimination (Base model) | 1074.458 | - | - |
| 2 | Model 1+WT on CL | 1027.463 | -46.9949 | < 0.001 |
| 3 | Model 2+Age on V_d_ | 998.839 | -28.624 | < 0.001 |
| 4 | Model 3+WT on V_d_ | 982.931 | -15.908 | < 0.001 |
| 5 | Model 4+SCr on CL (Full model) | 971.000 | **-11.931** | **< 0.001** |
| 6 | Model 5-WT on V_d_ | 987.532 | **+16.532** | **< 0.001** |
| 7 | Model 5-Age on V_d_ | 971.014 | +0.014 | > 0.05 |
| 9 | Model 5-WT on CL | 1067.599 | **+80.067** | **< 0.001** |
| 10 | Final model ^b^ | 971.014 | -103.444 | < 0.001 |

Abbreviations: OFV, objective function value; △OFV, the change of objective function value; WT, body weight (kg); SCr, serum creatinine (μmol/L); CL, clearance; V_d_, volume of distribution.

^a^ During forward selection, a covariate would be retained if a decrease in objective function value (OFV) was >3.84 after addition to the basic model. A more stringent criterion was used for the backward elimination step, where a covariate was independently removed from the full model if the increase in OFV was <10.83.

^b^ Body weight and creatinine was identified as significant covariates (values in boldface).

TABLE S4 Comparison of the teicoplanin dosing regimen in prescription before and after the update in Chinese.

| Population | Severity of infection | Dosing regimen in prescription | |
| --- | --- | --- | --- |
|  |  | Before update | After update |
| Adult and elderly patients with normal renal function | Moderate infection | Loading dose: 400 mg/d every 12h for 3 doses  Maintenance dose: **200** mg/d once daily | Loading dose: 400 mg/d every 12h for 3 doses  Maintenance dose: **400** mg/d once daily |
|  | Severe infection | Loading dose: **400** mg/d every 12h for **3** doses  Maintenance dose: **400** mg/d once daily | Loading dose: **800** mg/d every 12h for **3 to 5** doses  Maintenance dose: **800** mg/d once daily |
| Adult and elderly patients with normal renal function | - | Dose adjustment is not required until the fourth day of treatment, at which time dosing should be adjusted according to the result obtained from therapeutic drug monitoring (TDM) | Dose adjustment is not required until the fourth day of treatment, at which time dosing should be adjusted according to the result obtained from TDM |
| Children (2 months to 12 years) | Moderate infection | Loading dose: **10** mg/kg every 12h for 3 doses  Maintenance dose: **6** mg/kg once daily | Loading dose: **10** mg/kg every 12h for 3 doses  Maintenance dose: **6 – 10** mg/kg once daily |
|  | Severe infection | Loading dose: 10 mg/kg every 12h for 3 doses  Maintenance dose: 10 mg/kg once daily |  |
| Neonates and infants up to the age of 2 months | - | Loading dose: 16 mg/kg for 1 dose  Maintenance dose: 8 mg/kg once daily | Loading dose: 16 mg/kg for 1 dose  Maintenance dose: 8 mg/kg once daily |

**
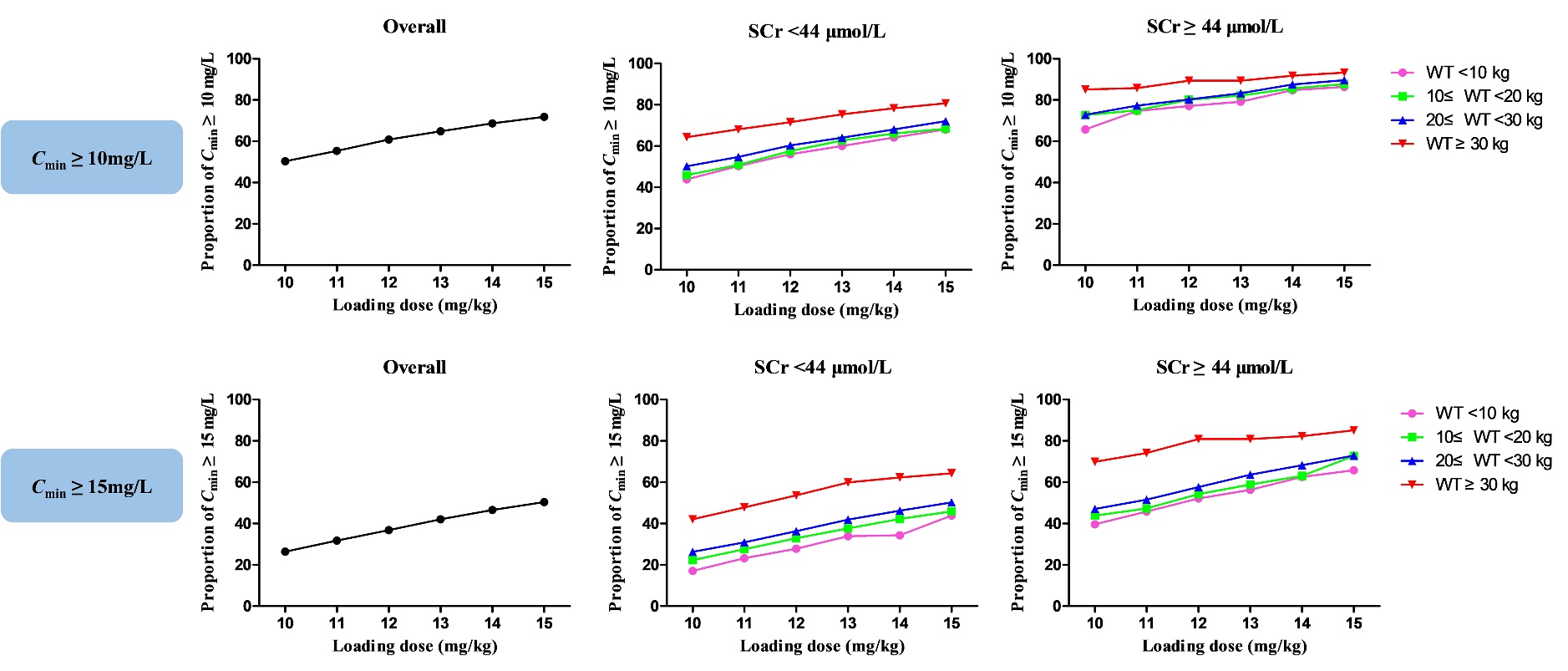
FIGURES**

**FIGURE S1** Proportion of patients achieving *C*_min_ ≥10 mg/L (moderate infection) and 15 mg/L (severe infection) for different loading doses in all simulated population (n =5000) and subgroups stratified by body weight (WT, kg) and serum creatinine (SCr, μmol/L). Loading doses were administered every 12 h for three doses and *C*_min_ was simulated by day 3 (48 h).

**
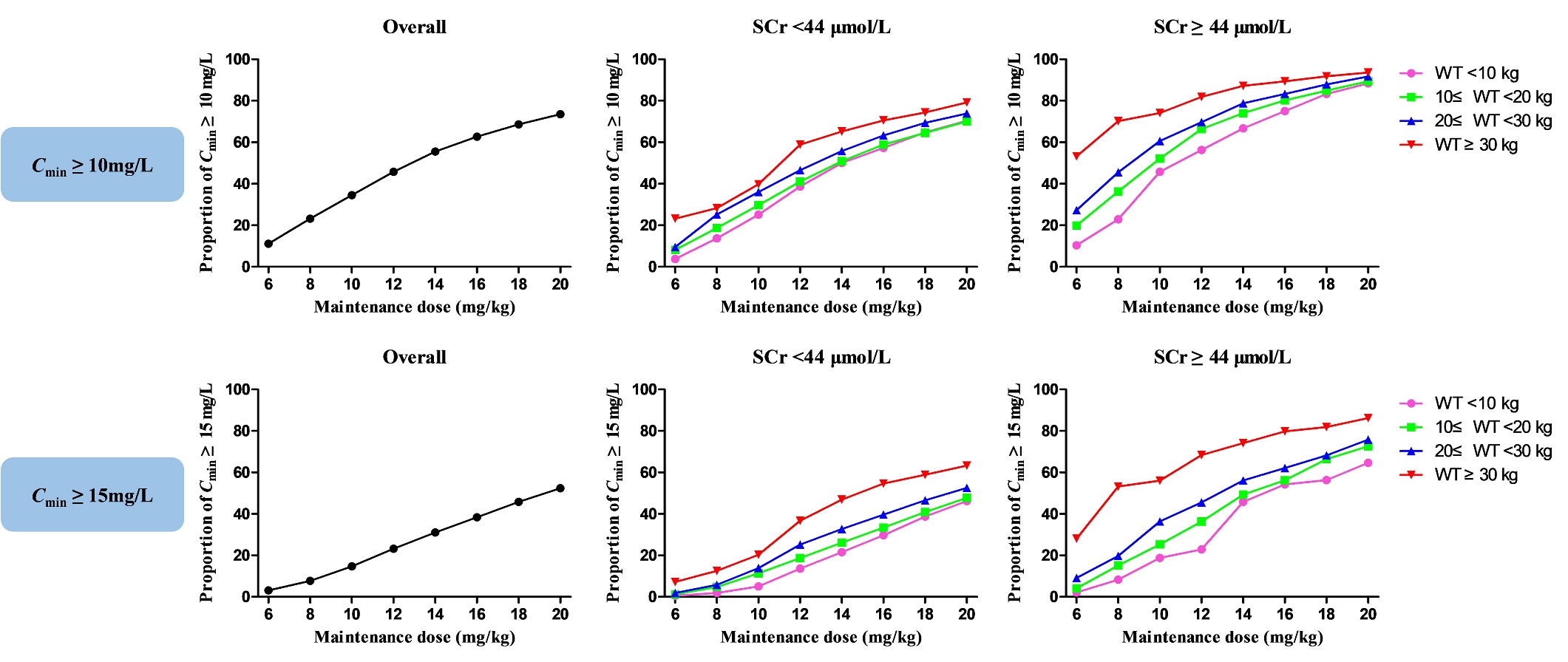
FIGURE S2** Proportion of patients achieving *C*_min_ ≥10 mg/L (moderate infection) and 15 mg/L (severe infection) for different maintenance doses in all simulated population (n =5000) and subgroups stratified by body weight (WT, kg) and serum creatinine (SCr, μmol/L). Maintenance doses were administered once daily and *C*_min_ was simulated by day 5 (96 h).

A standard loading dose of 10 mg/kg provided 50.4% and 26.4% of patents achieving 10 and 15 mg/L, respectively (Figure S1). Similarly, with standard maintenance doses of 6–10 mg/kg/day, only 11.1%–34.5% and 3.1%–14.7% of patients achieved target *C*_min_ of 10 and 15 mg/L, respectively (Figure S2). Poor probability of target attainment associating with lower WT and SCr was also observed. Even though with higher maintenance doses as we suggest (12 and 16 mg/kg/day), only 45.8% and 38.4% of patients reach the target *C*_min_ of 10 and 15 mg/L, respectively.
